# Knowledge, attitudes, prevention and opinion of nursing professionals regarding COVID-19: analytical study^*^

**DOI:** 10.1590/1518-8345.7412.4416

**Published:** 2025-01-31

**Authors:** Claudia Consuelo Torres-Contreras, Moisés Alfonso Bravo-Gómez, Raquel Rivera-Carvajal, Mario Castillo-Blanco, Diana Isabel Cáceres-Rivera

**Affiliations:** 1 Universidad de Santander, Facultad Ciencias de la Salud, Instituto de Investigacion Masira, Bucaramanga, Santander, Colombia.; 2 Asociación de Medicina Crítica y Cuidado Intensivo, Bucaramanga, Santander, Colombia.; 3 Universidad Cooperativa de Colombia, Facultad de Enfermería, Bucaramanga, Santander, Colombia.

**Keywords:** COVID-19, Learning, Intensive Care Units, Nursing Research, Advanced Practice Nursing, Critical Care Nursing

## Abstract

**Objective:**

to analyze the relationship between the knowledge, prevention, attitudes and opinion of nursing professionals in adult intensive care units regarding COVID-19 and their sociodemographic and work characteristics.

**Method:**

cross-sectional, analytical study. 124 nursing professionals who worked in adult intensive care units after the COVID-19 pandemic were included. To measure the variables, the Awareness, Attitudes, Prevention and Perceptions of COVID-19 Outbreak among Nurses questionnaire was used. To identify differences between the groups, the following tests were used: Kruskal-Wallis and Mann-Whitney U, Pearson correlation, and multiple analysis in logistic regression for each dimension.

**Results:**

participants with a workplace in private institutions showed a tendency towards better scores in awareness OR=3.92 (95%CI:1.50; 10.25), in prevention OR=8.93 (95%CI:3.12; 25.565), in attitude OR=2.77 (95%CI: 1.16; 6.58) and in perception with an OR= 19.65 (95%CI: 5.85; 65.94). In attitude, male participants showed a better result with OR=3.31 (95%CI: 1.18; 9.23) and in relation to perception, those who showed the best results were those with postgraduate studies as specialists OR=7.60 (95%CI: 1.73; 33.23).

**Conclusion:**

working in a private institution and having a postgraduate specialization degree were related to better scores in the dimensions of the scale.

## Highlights

**(1)** Years of experience presented a positive correlation with knowledge.

**(2)** High levels of awareness, practices, prevention and opinions were obtained.

**(3)** Professionals with specialization have higher COVID-19 KAB scores.

**(4)** Older nursing professionals tended to have lower scores in the dimensions of the instrument (knowledge, prevention, attitude and opinion).

**(5)** The pandemic period improves knowledge and practices in nursing.

## Introduction

Nursing professionals played a crucial role in the fight against the COVID-19 pandemic. They were fundamental in direct care of infected patients, in supporting the implementation of prevention measures, community education and in many other aspects^(1)^. Their knowledge, attitudes and opinions have evolved during and after the pandemic thanks to the rapid expansion of scientific knowledge about the virus and the disease^(2)^.

Regarding the knowledge of health professionals and nursing staff, in particular, they have had to constantly stay updated on care protocols, best practices in infection prevention and new research related to COVID-19^(3)^, including a central issue such as vaccination^(4)^. Regarding prevention, they have been involved in the implementation of measures, such as the proper use of personal protective equipment (PPE) and the promotion of vaccination. Work has also been evident in educating patients, families and communities about the importance of these practices to reduce the spread of this virus and its variants^(5-8)^.

In this same sense, the pandemic generated a considerable emotional burden for nursing professionals where the high level of commitment and dedication was recognized despite the physical and emotional challenges involved in working in highly stressful and often dangerous environments, this being evident in the attitudes that professionals have taken regarding the care of these patients and their families^(9-12)^.

On the other hand, some professionals have expressed concerns about the lack of resources and support, as well as burnout as persistent negative aspects after the pandemic^(13-14)^, while others have highlighted the importance of solidarity and teamwork as lessons learned^(15)^.

Lessons learned from the management of SARS COV-1 in 2003 suggest that knowledge of and attitudes towards infectious diseases are associated with the level of panic, an emotion that can complicate attempts to prevent and avoid the spread of contagion of an infectious disease^(16)^.

The KAB model (Knowledgement, Attitudes, and Behavior) is a theoretical model in education that explains how knowledge influences behavioral changes and emphasizes that these are the product of the interaction between knowledge and attitudes. This model establishes that health behavior can be modified by three continuous change processes: acquisition of knowledge, formation of beliefs and development of behaviors^(6,17)^.

Attitudes, knowledge and practices can play an important role in the way control measures are accepted to prevent an infectious disease and its spread and adherence to care in general, as is the case with measures for COVID-19^(18)^. Additionally, it is necessary to take into account that confinement demonstrated behavioral, somatic and affective changes in nursing staff, with which the evaluation of the processes of learning, knowledge and exercise of the profession increasingly take on greater relevance^(19)^.

Nursing professionals may have different opinions regarding infectious diseases and, therefore, may apply different clinical management strategies, leading to different patient outcomes^(20)^. On the other hand, practices are behaviors during interaction with patients as prevention and care practices for patients with this diagnosis.

For this reason, the contribution of nursing professionals during and after the pandemic has been essential to combat and prevent the spread of the virus and its variants, as well as to provide quality care to those affected by this disease in the short and long term^(16-18)^. That is why the research team set out to analyze the relationship between the knowledge, prevention, attitudes and opinions of nursing professionals in intensive care units against COVID-19 with use of the instrument Awareness, Attitudes, Prevention and Perceptions of COVID-19 Outbreak among Nurses^(21)^ and their sociodemographic and work characteristics in the Santander region, Colombia.

## Method

### Study design

Observational, cross-sectional, analytical study with information from nursing professionals who worked in Adult Intensive Care Units (IACU) during 2023 in Santander, Colombia.

### Population and sample

The required sample size was considered to be 124 nurses, taking into account an increase of 15% due to possible non-responses, admitting an alpha error of 0.05; a power of 0.80; a design effect of 1, the percentages of low levels reported for the dimensions were taken as a basis, where the prevention dimension reports 7.6% and would be the one that requires the largest sample size, according to what was published by a study carried out carried out in Saudi Arabia in 2020^(21)^.

### Study instruments

Awareness, Attitudes, Prevention and Perceptions of COVID-19 Outbreak among Nurses developed by Al-Dossary, et al.^(19)^ was used, and consists of 4 sections: knowledge, prevention, attitudes and opinion, with 63 items with Cronbach’s alpha reported by the author of 0.73 to 0.97 in the dimensions. This instrument identifies levels as low, medium and high in each of its dimensions, based on Likert-type scale scores (1= Totally disagree to 5= Totally agree). For the present study, a Cronbach’s alpha was obtained in the knowledge dimension of 0.90, in prevention of 0.96, in attitude of 0.80 and in opinion of 0.83 respectively.

The instrument was translated and back-translated, the facial and content validation of the questionnaire was carried out with the concept of 11 experts selected according to the criteria of the Fehring model^(22)^ with evaluation of clarity, precision, pertinence and relevance, scores were obtained content validity index (CVI) with scores greater than 0.8 in 55 of the 63 items, items with CVI less than 0.8 corresponded to:

*Knowledge dimension*: item 5. COVID-19 is transmitted through close contact with infected animals; Item 13. COVID-19 has a lower mortality rate than H1N1 respiratory infection; Item 19. COVID-19 can be transmitted by eating raw or undercooked meat or animal organs; Item 23. There is currently no vaccine available against COVID-19; Item 26. Current therapeutic strategies to treat COVID-19 infection are only preventive and supportive; Item 30. People who have had close contact with a confirmed COVID-19 positive patient during the 14 days prior to the onset of symptoms should be quarantined and undergo a diagnostic test for COVID-19; item 32. If the test result is positive, it is recommended that the test be repeated for verification.

*Prevention dimension*: item 10. Only suspected cases of patients with COVID-19 should be kept in quarantine.

*Attitude dimension*: item 6. Having monetary compensation from the government or my workplace reduces my anxiety regarding the risk of contracting the infection.

The authors of the validation of the instrument determined to delete items 5 and 19 from the knowledge dimension, item 10 from the prevention dimension, and item 6 from the attitude dimension. The other items of the knowledge dimension were left for the analysis and the scores of items 13, 23 and 32 were reversed respectively. The validated instrument was then left with 36 items in the knowledge dimension, 13 in prevention, 5 in attitude and 4 in opinion, for a total of 58 items.

The levels are presented based on the averages of the Likert scale scores, where scores of 1 to 1.79 are very low, between 1.8 to 2.59 are low, between 2.6 to 3.39 are medium, between 3.4 to 4.19 high and from 4.2 to 5 very high^(23)^.

### Data collection

The data was collected from February to May 2023, the application was submitted online. With information from the databases of the intensive care critical medicine association for Santander, stratified sampling was carried out selecting nursing professionals who currently work in the intensive care units that care for patients with a positive diagnosis for COVID-19 in the municipalities from: Bucaramanga, Floridablanca, Piedecuesta, San Gil, Socorro and Barrancabermeja. As inclusion criteria, it was considered that they had been working for more than 3 months in the IACU where patients with a diagnosis of COVID-19 are treated. Physical disability or not having access to technology to answer the questionnaire were considered exclusion criteria.

### Data analysis

In the analysis, the qualitative measurement variables were described with absolute and relative frequencies, the continuous variables were described with measures of central tendency and dispersion according to the distribution, with normal distribution the mean and standard deviation were presented, and the nonparametric variables were presented with medians and interquartile ranges; the Shapiro-Francia test was used to verify the distribution.

A bivariate analysis was performed with the mean scores in each of the dimensions, to compare the characteristics of the participants, using the respective Kruskal-Wallis statistical tests and Mann-Whitney U test. The correlation analysis was performed with Pearson’s statistics for the numerical variables after evaluating the normal distribution and a graph was generated in the R software. For the multiple analysis, logistic regression was used to determine the probability of being in the highest level in the comparison. An assessment of the model’s fit was performed for the other levels. The calculations were performed in the statistical software Stata v17. The database and analysis of the instrument validation process and the results presented were loaded into Mendeley Data^(24)^.

### Ethical aspects

The study was based on Resolution 8430 of 1993 of the Colombian Ministry of Health^(25)^ and the Declaration of Helsinki promulgated by the World Medical Association in 2000^(26)^. The research was considered a risk-free study. Among the ethical principles of the research, autonomy, confidentiality, beneficence and non-maleficence were taken into account and informed consent was requested, which was linked in the online form before the instrument; In the document, the objective of the research was explained to the participants and it was highlighted that their participation and withdrawal were voluntary. The present study was endorsed by the bioethics committees of the University of Santander, with registration No. 004 of February 15, 2022.

## Results

Regarding the characteristics of the 124 study participants, the majority identified a young population with 46.77% (58/124) in an age range between 25 and 30 years, followed by participants between 31 and 35 years old with 30.65% (38/124), those over 46 years of age corresponded to 3.23% (4/124), 76.61% (95/124) were female and 23.39% were male (29/124), the marital status with the highest prevalence was single with 69.35% (86/124), followed by married with 29.03% (36/124). In the years of work experience in the IACU, a median of 7 (IR: 4; 10.5) years was identified, the number of work hours per day with a median of 12 (IR: 12; 12). Regarding the educational level, it was identified that 77.42% (96/124) corresponded to professionals with an undergraduate degree, 18.55% (23/124) to specialists and 4.03% (5/124) to professionals with a master’s degree. With links to health institutions in the public sector 42.98% (49/124) and 57.02% (65/124) in the private sector. The dimension with the best median score was prevention, with a median of 4.73 (4.07; 5) and the lowest was opinion with a median of 4 (4; 4.75) (Table 1).

When comparing the median scores of the dimensions according to sociodemographic characteristics and occupations, differences are seen with values in the dimensions of knowledge, prevention and opinion where participants who work in private institutions have higher medians than those in public areas (p <0.05). In prevention and attitude, male participants presented higher medians compared to female participants (Table 1).

**Table 1:** - Characteristics of the participants. Medians and interquartile ranges according to dimensions (n = 124). Santander, Colombia, 2023

**Feature**	**%(n)**	**Knowledge**			**Prevention**			**Attitude**			**Opinion**		
	100(124)	4.36	(4; 4.58)		4.73	(4.07; 5)		4.20	(4; 5)		4	(4; 4.75)	
Age in years				0.5795			0.6931			0.8182			0.1364
25-30	46.77(58)	4.36	(4.04; 4.58)		4.69	(4.07; 5)		4.20	(3.60; 5)		4	(3.75; 4.50)	
31-35	30.65(38)	4.27	(3.91; 4.55)		4.80	(4; 5)		4.40	(4; 4.80)		4.37	(4; 4.75)	
36-40	8.87(11)	4.47	(3.88; 4.63)		5	(4.07; 5)		4.60	(3,20; 5)		4	(3.75; 5)	
41-45	10.48(13)	4.38	(3.94; 4.55)		4.38	(4.07; 4.92)		4	(4; 4.40)		4.25	(4; 5)	
> 46	3.23(4)	4.47	(4.31; 4.68)		4.92	(4.61; 5)		4.60	(4; 5)		4.85	(4,12; 5)	
Sex				0.2397			**0,0016**			**0.0525**			0.2965
Female	76.61(95)	4.29	(3.97; 4.58)		4.69	(4.62; 4.92)		4.20	(4; 4.80)		4	(4; 4.75)	
Male	23.39(29)	4.50	(4.22; 4.61)		5	(4.38; 5)		4.80	(4,20; 5)		4.50	(4; 5)	
Marital status				0.9508			0.0941			0.2618			0.1399
Single	69.35(86)	4.36	(3.97; 4.58)		4.73	(4.07; 5)		4.20	(4; 5)		4	(4; 4.50)	
Married	29.03(36)	4.37	(4.02; 4.58)		4.80	(4,15; 5)		4.20	(4; 4.80)		4.50	(4; 5)	
Divorced	1.61(2)	4.30	(4.22; 4.38)		2.57	(1.15; 4)		2.90	(1.80; 4)		4.37	(3.75; 5)	
Educational level				0.3186			0.5817			0.2343			0.2767
Professional	77.42(96)	4.27	(3.97; 4.58)		4.69	(4.07; 5)		4.20	(4; 5)		4	(4; 4.75)	
Specialist	18.55(23)	4.50	(4.25; 4.63)		4.84	(4,15; 5)		4.60	(4; 5)		4.25	(4; 4.75)	
Master’s degree	4.03(5)	4.37	(4.34; 4.41)		4.92	(4.69; 4.92)		4.20	(3.60; 4.60)		5	(4.5; 5)	
Workplace				**0.0199**			**0.0166**			0.4375			**<0.0001**
Public	42.98(49)	4.22	(3.94; 4.55)		4.07	(4; 5)		4	(4; 4.80)		4	(3.75; 4)	
Private	57.02(65)	4.41	(4.22; 4.61)		4.76	(4.38; 5)		4.20	(4; 4.80)		4.50	(4; 5)	

Note: p-value: Kruskal-Wallis and Mann-Whitney U test

**Figure 1:**
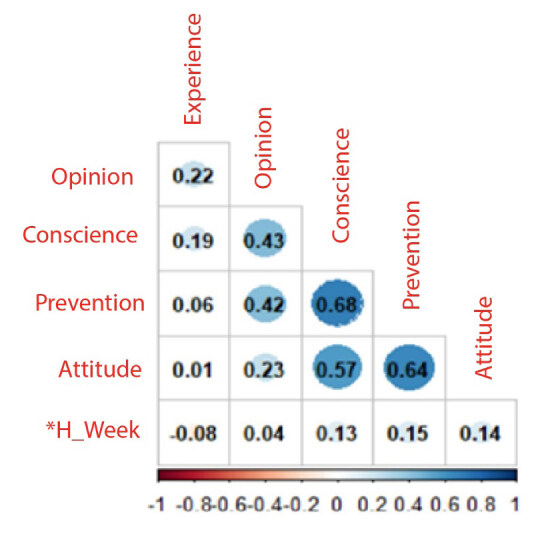
- Pearson correlation between numerical variables (n = 124). Bucaramanga, Colombia, 2023

Note: Experience in years

In the correlation analysis for the numerical variables, it was found that years of experience have a significant correlation with the number of working hours per day and the knowledge dimension. A correlation was also identified between the scores of the instrument dimensions, where the strongest ones correspond to the knowledge-prevention, prevention-attitude scores with correlations greater than 0.64 (P value <0.0001); Experience in years presented a significant correlation but with low intensity with knowledge-opinion, of 0.19 and 0.22 respectively (p value = 0.0398 and 0.0159); work hours per week were not significantly correlated with the linked variables (Figure 1).

*H_Week = Hours of work per week

**Figure 2:**
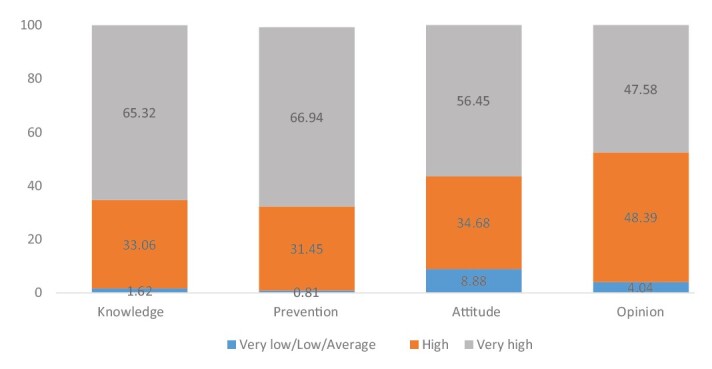
- Percentage distribution of levels according to instrument dimensions (n = 124). Bucaramanga, Colombia, 2023

In the analysis according to the categories, it can be seen that the dimensions have the highest percentage of very high and high levels of knowledge, prevention and attitude, where in knowledge 65.32% (81/124) with very high scores, 33. 06% (41/124) in high and the remaining 1.62% (2/124) with very low/low/medium levels, in prevention 66.94% (82/124) with high levels, 31 .45% (39/124) with high levels, and 0.81% (2) with very low/low/medium levels, for the attitude dimension 56.45% (70/124) with very high levels, the 34.68% (43/124) with high levels and 8.88% (11/124) with very low/low/medium levels. For the opinion dimension, 47.58% (59/124) with very high scores, 48.39% (60/124) with high scores, and 4.04% (5/124) with very low/low/medium levels (Figure 2).

In the multiple analysis, it is identified that nurses who work in private institutions have significantly (p value <0.05) scores in the very high categories in the different dimensions compared to those who work in public institutions. On the other hand, it seemed that having a level of specialization also favors very high scores. For the male gender, it can be seen that the attitude dimension is related to having very high scores (Table 2). In terms of age, a tendency was observed to have lower scores in the dimensions as the age range increased, and this was significantly so in the dimensions of knowledge and prevention for the category of 31 to 35 years old compared to those aged 25 to 30 years (Table 2).

**Table 2:** - Odds Ratio adjusted to be at the highest level in each of the dimensions of the scale (n = 124). Santander, Colombia, 2023

**Feature**	**Knowledge**	**Prevention**	**Attitude**	**Opinion**
Age in years				
25-30	1	1	1	1
31-35	0.23 (0.07, 0.78)*	0.20 (0.05, 0.77)*	1.12 (0.38, 3.22)	1.18 (0.34, 4.01)
36-40	0.12 (0.01, 1.09)	0.21 (0.02, 2.16)	0.85 (0.12, 6.05)	0.26 (0.03, 2.18)
≥ 41 years	0.13 (0.00, 1.90)	0.06 (0.00, 1.13)	0.28 (0.02, 2.83)	0.85 (0.06, 10.78)
Sex				
Female	1	1	1	1
Male	2.32 (0.73, 7.39)	2.73 (0.78, 9.55)	3.31 (1.18; 9.23)*	1.20 (0.41, 3.48)
Marital status				
Single	1	1	1	1
Married	0.93 (0.34, 2.53)	1.24 (0.43, 3.59)	1.01 (0.41, 2.48)	1.78 (0.61, 5.19)
Educational level				
Professional	1	1	1	1
Specialist	2.98 (0.79, 11.15)	2.36 (0.64, 8.63)	2.84 (0.89, 9.06)	7.60 (1.73; 33.23)^†^
Workplace				
Public	1	1	1	1
Private	3.92 (1.50, 10.25)	8.93 (3.12; 25.565)^‡^	2.77 (1.16; 6.58)*	19.65 (5.85; 65.94)^‡^

*p value <0.05; ^†^<0.01; ^‡^< 0.001

## Discussion

This study analyzed the relationship between awareness, prevention, attitudes and perception about COVID-19 with the sociodemographic and work characteristics of nursing professionals, where the dimensions of awareness and prevention presented percentages greater than 90%. This reflects the improvement in knowledge and the development of protocols in new infectious diseases, since during the critical period of the pandemic, there was a need to advance in the management of the disease^(27-29)^.

The above has been demonstrated in different studies carried out in leading countries such as China where adequate attitudes and practices have been reported in 97.40% and 92.70% respectively in the participants^(30)^. However, in relation to knowledge, only 6.40% obtained a good level, something disturbing, since the study was carried out two years after the start of the pandemic. Regarding the correlation between knowledge, attitudes and practices, *r scores* between 0.03 and 0.29 were obtained, which corresponds to lower scores than those reported in the present study^(30)^. These differences could be due to the efforts and different strategies used in the two countries where cultural differences and health educational processes are different.

The strong positive correlation identified between awareness-prevention, awareness-attitude and prevention-attitude with an ***r*** greater than 0.50, as well as between the years of experience with the awareness dimension with an r=0.19 and the years of experience with The opinion dimension with an *r* =0.19, is similar to what was described in a study carried out with 182 IACU nurses in Jordan where they identified a relationship between years of experience and knowledge^(31)^. Likewise, in Saudi Arabia, a relationship was found between better knowledge scores with a higher educational level, with specialists being those with the best levels^(32)^. These findings show the effect and need for continuing education programs for nursing professionals^(33-34)^.

Regarding the type of institution where one works, that is, public or private, it could be related to the availability of information, institutional protocols and practices, training and individual experience, among other aspects. However, it is something that must continue to be evaluated since the studies found correspond to different contexts where culture and politics can vary radically^(35-36)^.

Regarding gender, it was identified that male participants presented better scores, especially in the attitude dimension. It should be considered that this could be due to the number of nurses who participated, which corresponds to a lower percentage of the sample. In relation to attitudes and their differences between men and women, a study was found about the reasons for changing their job or area of performance and they had to do with reasons mainly of economic remuneration^(37)^.

As contributions to knowledge, this study identifies the validation and use of a structured instrument for Colombia, which allowed evaluating the dimensions of awareness, attitudes, prevention and practices for COVID-19. Furthermore, the strength of the study is the sample size, as well as the multiple analysis.

Among the limitations can be considered the population that includes only service personnel of the ICU and the type of sampling, given that performing a census would provide greater power in the calculations^(38)^, as well as the context and significant number of patients in the ICU with respect to the time of the pandemic and data collection, It should be noted that respiratory infection by COVID-19 is a pathological entity that is still in the process of study, which implies that knowledge about this disease is constantly evolving.

For future studies, the relationship between levels of the different dimensions of the scale with anxiety variables^(29)^, personal and family pathological history related to COVID-19^(31)^; on the other hand, it is important to highlight the rapid evolution in everything related to COVID-19, where the importance of updating is identified to provide care, have more precise diagnoses and effective, efficient and effective treatments, as well as innovative promotion, advancement and prevention measures^(39-42)^.

## Conclusion

The levels of awareness, prevention, attitude and opinion around COVID-19 presented better scores when the professionals worked in private institutions and when the educational level was higher. Furthermore, in the attitude dimension, better scores were seen in male participants.
