# Supplementary material for: Knowledge, attitudes, prevention and opinion of nursing professionals regarding COVID-19: analytical study
Source: Rev Lat Am Enfermagem. 2025 Jan 31;33:e4416. doi: 10.1590/1518-8345.7412.4416 (PMC11789758; doi:10.1590/1518-8345.7412.4416)
Supplement: Translation

# Translation: Conocimientos, actitudes, prevención y opinión de los profesionales de enfermería frente al COVID-19: estudio analítico^*^

**DOI:** 10.1590/1518-8345.7412.4415

**Authors:** Claudia Consuelo Torres-Contreras, Moisés Alfonso Bravo-Gómez, Raquel Rivera-Carvajal, Mario Castillo-Blanco, Diana Isabel Cáceres-Rivera

**Affiliations:** 1 Universidad de Santander, Facultad Ciencias de la Salud, Instituto de Investigacion Masira, Bucaramanga, Santander, Colombia.; 2 Asociación de Medicina Crítica y Cuidado Intensivo, Bucaramanga, Santander, Colombia.; 3 Universidad Cooperativa de Colombia, Facultad de Enfermería, Bucaramanga, Santander, Colombia.

**Keywords:** COVID-19, Aprendizaje, Unidades de Cuidados Intensivos, Investigación en Enfermería, Enfermería de Práctica Avanzada, Enfermería de Cuidados Críticos

## Abstract

**Objetivo:**

analizar la relación entre el conocimiento, prevención, actitudes y opinión de los profesionales de enfermería en unidades de cuidado intensivo adulto frente al COVID-19 y sus características sociodemográficas y laborales.

**Método:**

estudio transversal, analítico. Se incluyeron 124 profesionales de enfermería que laboraron en Unidades de Cuidado intensivo adulto posterior a la pandemia por COVID-19. Para la medición de las variables se utilizó el cuestionario *Awareness, Attitudes, Prevention and Perceptions of COVID-19 Outbreak among Nurses*; para identificar diferencias entre los grupos se utilizaron las pruebas: Kruskal-Wallis y U de Mann-Whitney, se realizó correlación de Pearson, análisis múltiple en regresión logística para cada una dimensión.

**Resultados:**

los participantes con lugar de trabajo en instituciones privadas presentaron tendencia a mejores puntajes en conciencia OR=3.92(IC95%:1.50; 10.25), en prevención OR=8.93(IC95%:3.12; 25.565), en actitud OR=2.77(IC95%:1.16; 6.58) y en percepción con un OR=19.65(IC95%:5.85; 65.94). En actitud, los participantes de género masculino mostraron un mejor resultado con OR=3.31(IC95%:1.18; 9.23) y con relación a la percepción quienes mostraron mejor resultado fueron aquellos con estudio de posgrado como especialistas OR=7.60(IC95%:1.73; 33.23).

**Conclusión:**

el desempeñarse laboralmente en una institución privada y tener un posgrado tipo especialización se relacionaron con mejores puntajes en las dimensiones de la escala.

## Destacados

**(1)** Los años de experiencia presentaron una correlación positiva con los conocimientos.

**(2)** Se obtuvieron niveles altos de conciencia, prácticas, prevención y opiniones.

**(3)** Los profesionales con especialización tienen mayores puntuaciones en KAB de la COVID-19.

**(4)** Los profesionales de enfermería con mayor edad presentaron tendencia a puntajes más bajos en las dimensiones del instrumento (conocimiento, prevención, actitud y opinión).

**(5)** El periodo de la pandemia mejora los conocimientos y las prácticas en enfermería.

## Introducción

Los profesionales de enfermería desempeñaron un papel crucial en la lucha contra la pandemia de COVID-19. Fueron fundamentales en la atención directa a pacientes infectados, en el apoyo a la implementación de medidas de prevención, educación a la comunidad y en muchos otros aspectos^(1)^. Sus conocimientos, actitudes y opiniones han evolucionado durante y posterior a la pandemia gracias a la rápida expansión del conocimiento científico sobre el virus y la enfermedad^(2)^.

Con relación a los conocimientos los profesionales de la salud y personal de enfermería, en particular, han tenido que mantenerse actualizados constantemente sobre los protocolos de atención, las mejores prácticas en prevención de infecciones y las nuevas investigaciones relacionadas con el COVID-19^(3)^, incluido un tema central como es la vacunación^(4)^. En lo que respecta a la prevención, han estado involucrados en la implementación de medidas, como el uso adecuado de equipos de protección personal (EPP) y la promoción de la vacunación. También se ha evidenciado el trabajo en la educación a pacientes, familias y comunidades sobre la importancia de estas prácticas para reducir la propagación de este virus y sus variantes^(5-8)^.

En este mismo sentido, la pandemia generó una carga emocional considerable para los profesionales de enfermería en donde se reconoció el alto nivel de compromiso y dedicación a pesar de los desafíos físicos y emocionales que implica trabajar en entornos altamente estresantes y a menudo peligrosos, siendo esto evidente en las actitudes que los profesionales han tomado respecto a la atención de estos pacientes y sus familias^(9-12)^.

Por otro lado, algunos profesionales han expresado preocupaciones sobre la falta de recursos y apoyo, así como el agotamiento como aspectos negativos persistentes después de la pandemia^(13-14)^, mientras que otros han destacado la importancia de la solidaridad y el trabajo en equipo como lecciones aprendidas^(15)^.

Las lecciones aprendidas del manejo del SARS-COV-1 en 2003 sugieren que el conocimiento y las actitudes con las enfermedades infecciosas están asociadas con el nivel de pánico, emoción que puede complicar los intentos de prevenir y evitar la propagación del contagio de una enfermedad infecciosa^(16)^.

El modelo KAB (*Knowledgement, Attitudes, and Behavior*) es un modelo teórico en educación que explica cómo influye el conocimiento en los cambios comportamentales y enfatiza que estos son producto de la interacción entre el conocimiento y las actitudes. Este modelo establece que el comportamiento en salud puede ser modificado por tres procesos de cambio continuos: adquisición de conocimiento, formación de creencias y desarrollo de comportamientos^(6,17)^.

Las actitudes, conocimientos y prácticas pueden jugar un rol importante en la manera como se aceptan las medidas de control para prevenir una enfermedad infecciosa y su diseminación y la adherencia al cuidado en general, como lo es en el caso de las medidas para el COVID-19^(18)^. Adicionalmente, es necesario tener en cuenta que el confinamiento demostró cambios conductuales, somáticos y afectivos en el personal de enfermería, con lo cual, la evaluación de los procesos de aprendizaje, conocimiento y ejercicio de la profesión cada vez toman una mayor relevancia^(19)^.

Los profesionales de enfermería pueden tener diferentes opiniones frente a las enfermedades infecciosas y, por lo tanto, pueden aplicar diferentes estrategias de manejo clínico, lo que lleva a diferentes resultados en el paciente^(20)^. Por otro lado, las prácticas son los comportamientos durante la interacción con los pacientes como prácticas de prevención y cuidado a los enfermos con este diagnóstico.

Es por ello, que la contribución de los profesionales de enfermería durante y posterior a la pandemia han sido fundamental para combatir y prevenir la propagación del virus y sus variantes, así como para brindar atención de calidad a los afectados por esta enfermedad en el corto y largo plazo^(16-18)^. Es por ello que el equipo investigador se propuso analizar la relación entre el conocimiento, prevención, actitudes y opiniones de los profesionales de enfermería en unidades de cuidado intensivo frente al COVID-19 con uso del instrumento *Awareness, Attitudes, Prevention and Perceptions of COVID-19 Outbreak among Nurses* ^(21)^ y sus características sociodemográficas y laborales en la región de Santander, Colombia.

## Método

### Diseño del estudio

Estudio observacional, transversal, analítico con información de profesionales de enfermería que se desempañaban en Unidades de Cuidados Intensivos Adultos (UCIA) durante el 2023 en Santander, Colombia.

### Población y muestra

El tamaño de muestra requerido se consideró de 124 enfermeros(as), teniendo en cuenta un aumento del 15% por posibles no respuestas, admitiendo un error alpha de 0,05; un poder de 0,80; un efecto en el diseño de 1, se tomó como base los porcentajes de bajos niveles de reportados para las dimensiones, donde la dimensión de prevención reporta 7,6% y sería la que exige el mayor tamaño de muestra, según lo publicado por estudio llevado a cabo en Arabia Saudita en 2020^(21)^.

### Instrumentos del estudio

Se utilizó el Instrumento: *Awareness, Attitudes, Prevention and Perceptions of COVID-19 Outbreak among Nurses,* elaborado por Al-Dossary, et al.^(19)^, el cual consta de 4 secciones: conocimientos, prevención, actitudes y opinión, con 63 ítems con alpha de Cronbach reportada por el autor de 0,73 a 0,97 en las dimensiones. Este instrumento identifica los niveles como bajo, medio y alto en cada una de sus dimensiones, con base en los puntajes de escalas tipo Likert (1= Totalmente en desacuerdo a 5= Totalmente de acuerdo). Para el presente estudio se obtuvo un alpha de Cronbach en la dimensión de conocimiento de 0,90, en prevención de 0,96, en actitud de 0,80 y en opinión de 0,83 respectivamente.

El instrumento fue traducido y retro-traducido, la validación facial y de contenido del cuestionario se realizó con concepto de 11 expertos seleccionados según los criterios del modelo de Fehring^(22)^ con evaluación de la claridad, precisión, pertinencia y relevancia, se obtuvieron puntuaciones de índice de validez de contenido (IVC) con puntajes superiores a 0,8 en 55 de los 63 ítems, los ítems con IVC menores a 0,8 correspondieron a:

*Dimensión conocimiento:* ítem 5. El COVID-19 se transmite por contacto cercano con animales infectados; ítem 13. El COVID-19 tiene una tasa de mortalidad menor que la infección respiratoria por H1N1; ítem 19. El COVID-19 se puede transmitir al comer carne u órganos de animales crudos o poco cocidos; ítem 23. Actualmente no hay ninguna vacuna disponible contra el COVID-19; ítem 26. Las estrategias terapéuticas actuales para tratar la infección por COVID-19 son solo preventivas y de apoyo; ítem 30. Las personas que hayan tenido contacto cercano con un paciente confirmado con COVID-19 positivo durante los 14 días anteriores al inicio de los síntomas deben ser puestas en cuarentena y realizarse una prueba diagnóstica para COVID-19; ítem 32. Si el resultado de la prueba es positivo, se recomienda que se repita la prueba para su verificación.

*Dimensión prevención:* ítem 10. Solo los casos sospechosos de pacientes con COVID-19 deben mantenerse en cuarentena.

*Dimensión actitud:* ítem 6. Tener una compensación monetaria por parte del gobierno o mi lugar de trabajo, disminuye mi ansiedad con respecto al riesgo de contraer la infección.

Los autores de la validación del instrumento determinaron suprimir de la dimensión de conocimiento los ítems 5 y 19, de la dimensión de prevención el ítem 10 y de la dimensión de actitud el ítem 6. Los demás ítems de la dimensión de conocimiento se dejaron para el análisis y se invirtieron los puntajes de los ítems 13, 23 y 32 respectivamente. El instrumento validado quedo entonces con 36 ítems en la dimensión de conocimiento, 13 en prevención, 5 en actitud y 4 en opinión, para un total de 58 ítems.

Los niveles se presentan con base en los promedios de los puntajes de la escala Likert, donde puntajes de 1 a 1,79 son muy bajos, entre 1,8 a 2,59 son bajos, entre 2,6 a 3,39 son medios, entre 3,4 a 4,19 altos y de 4,2 a 5 muy altos^(23)^.

### Recolección de datos

Los datos fueron recolectados desde febrero a mayo de 2023, se envió la solicitud en línea. Con la información de las bases de datos de la asociación de medicina crítica de cuidado intensivo para Santander, se realizó muestreo estratificado seleccionando profesionales de enfermería que actualmente laboran en las unidades de cuidado intensivo que atienden pacientes con diagnóstico positivo para COVID-19 de los municipios de Bucaramanga, Floridablanca, Piedecuesta, San Gil, Socorro y Barrancabermeja. Como criterios de inclusión se consideró que llevaran más de 3 meses desempeñándose en UCIA donde se atienden pacientes con diagnóstico de COVID-19; como exclusión discapacidad física o no tener acceso a tecnológica para responder el cuestionario.

### Análisis de datos

En el análisis las variables de medición cualitativa se describieron con frecuencias absolutas y relativas, las variables continuas se describieron con medidas de tendencia central y de dispersión según la distribución, con distribución normal se presentaron promedio y desviación estándar y las no paramétricas con medianas y rangos, intercuartílico para verificar la distribución se utilizó la prueba de Shapiro-Francia.

Se realizó análisis bivariado con los puntajes medios en cada una de las dimensiones, para comparar las características de los participantes y haciendo uso de las pruebas estadísticas respectivas Kruskal-Wallis y prueba U de Mann-Whitney. Se realizó análisis de correlación con el estadístico de Pearson para las variables numéricas previa evaluación de la distribución normal y se generó gráfica en el *software* R. Para el análisis múltiple se utilizó regresión logística para determinar la probabilidad de estar en el nivel más alto en comparación con los demás niveles, se realizó evaluación del ajuste del modelo. Los cálculos se realizaron en el *software* estadístico Stata v17. La base de datos y el análisis del proceso de validación del instrumento y de los resultados presentados fueron cargados en *Mendeley Data* ^(24)^.

### Aspectos éticos

El estudio se basó en la Resolución 8430 de 1993 del Ministerio de Salud de Colombia^(25)^ y en la Declaración de Helsinki promulgada por la Asociación Médica Mundial en el año 2000^(26)^. La investigación se consideró un estudio sin riesgo. Entre los principios éticos de la investigación se tuvo en cuenta la autonomía, confidencialidad, beneficencia y no maleficencia y se solicitó el consentimiento informado, que fue vinculado en el formulario *on line* antes del instrumento; en el documento se explicó a los participantes el objetivo de la investigación y se destacó que su participación y retirada eran voluntarias. El presente estudio fue avalado por los comités de bioética de la Universidad de Santander, con registro No. 004 del 15 de febrero del año 2022.

## Resultados

Respecto de las características de los 124 participantes del estudio se identificó en su mayoría a una población joven con un 46,77% (58/124) en un rango de edad entre 25 a 30 años, seguida por participantes de 31 a 35 años con el 30,65% (38/124), los mayores de 46 años correspondieron al 3,23% (4/124), de sexo femenino el 76,61% (95/124) y de masculino el 23,39% (29/124), el estado civil con mayor prevalencia fue el soltero con el 69,35% (86/124), seguido por casados con el 29,03% (36/124). En los años de experiencia de trabajo en UCIA se identificó una mediana de 7 (RI: 4; 10,5) años, el número de horas de trabajo al día con mediana de 12 (RI: 12; 12). Respecto del nivel educativo se identificó que el 77,42% (96/124) correspondieron a profesionales con pregrado, el 18,55% (23/124) a especialistas y el 4,03% (5/124) a profesionales con maestría. Con vinculación a instituciones de salud del sector público el 42,98% (49/124) y del privado el 57,02% (65/124). La dimensión con mejor puntaje en la mediana fue la de prevención, con mediana de 4,73 (4.07; 5) y la menor fue la de opinión con mediana de 4 (4; 4,75) (Tabla 1).

Al comparar las medianas de los puntajes de las dimensiones según características sociodemográficas y ocupaciones, se aprecia diferencias con valores en las dimensiones de conocimientos, prevención y opinión donde los participantes que se desempeñan en instituciones privadas presentan medianas más altas que los de área pública (p<0,05); en la prevención y actitud los participantes de sexo masculino presentaron medianas más altas en comparación con las de género femenino (Tabla 1).

**Tabla 1:** - Características de los participantes. Medianas y rangos intercuartílico según dimensiones (n = 124). Santander, Colombia, 2023

**Característica**	**%(n)**	**Conocimiento**			**Prevención**			**Actitud**			**Opinión**		
	100(124)	4,36	(4; 4,58)		4,73	(4,07; 5)		4,20	(4; 5)		4	(4; 4,75)	
Edad en años				0,5795			0,6931			0,8182			0,1364
25-30	46,77(58)	4,36	(4,04; 4,58)		4,69	(4,07; 5)		4,20	(3,60; 5)		4	(3,75; 4,50)	
31-35	30,65(38)	4,27	(3,91; 4,55)		4,80	(4; 5)		4,40	(4; 4,80)		4,37	(4; 4,75)	
36-40	8,87(11)	4,47	(3,88; 4,63)		5	(4,07; 5)		4,60	(3,20; 5)		4	(3,75; 5)	
41-45	10,48(13)	4,38	(3,94; 4,55)		4,38	(4,07; 4,92)		4	(4; 4,40)		4,25	(4; 5)	
> 46	3,23(4)	4,47	(4,31; 4,68)		4,92	(4,61; 5)		4,60	(4; 5)		4,85	(4,12; 5)	
Sexo				0,2397			**0,0016**			**0,0525**			0,2965
Femenino	76,61(95)	4,29	(3,97; 4,58)		4,69	(4,62; 4,92)		4,20	(4; 4,80)		4	(4; 4,75)	
Masculino	23,39(29)	4,50	(4,22; 4,61)		5	(4,38; 5)		4,80	(4,20; 5)		4,50	(4; 5)	
Estado civil				0,9508			0,0941			0,2618			0,1399
Soltero/a	69,35(86)	4,36	(3,97; 4,58)		4,73	(4,07; 5)		4,20	(4; 5)		4	(4; 4,50)	
Casado/a	29,03(36)	4,37	(4,02; 4,58)		4,80	(4,15; 5)		4,20	(4; 4,80)		4,50	(4; 5)	
Divorciado/a	1,61(2)	4,30	(4,22; 4,38)		2,57	(1,15; 4)		2,90	(1,80; 4)		4,37	(3,75; 5)	
Nivel educativo				0,3186			0,5817			0,2343			0,2767
Profesional	77,42(96)	4,27	(3,97; 4,58)		4,69	(4,07; 5)		4,20	(4; 5)		4	(4; 4,75)	
Especialista	18,55(23)	4,50	(4,25; 4,63)		4,84	(4,15; 5)		4,60	(4; 5)		4,25	(4; 4,75)	
Maestría	4,03(5)	4,37	(4,34; 4,41)		4,92	(4,69; 4,92)		4,20	(3,60; 4,60)		5	(4,5; 5)	
Lugar de trabajo				**0,0199**			**0,0166**			0,4375			**<0,0001**
Público	42,98(49)	4,22	(3,94; 4,55)		4,07	(4; 5)		4	(4; 4,80)		4	(3,75; 4)	
Privado	57,02(65)	4,41	(4,22; 4,61)		4,76	(4,38; 5)		4,20	(4; 4,80)		4,50	(4; 5)	

Nota: Valor p: prueba Kruskal-Wallis y U de Mann-Whitney

 En el análisis de correlación para las variables numéricas se encontró que los años de experiencia tienen correlación significativa con la cantidad de horas de trabajo al día y la dimensión de conocimientos. También se identificó correlación entre los puntajes de las dimensiones del instrumento, donde aquellas más fuertes corresponden a los puntajes de conocimiento-prevención, prevención-actitud con correlaciones mayores a 0,64, (Valor p <0,0001); la experiencia en años presento correlación significativa pero con baja intensidad con conocimiento-opinión, de 0,19 y 0,22 respectivamente (valor p= 0,0398 y 0,0159); las horas de trabajo a la semana no se correlacionaron significativamente con las variables vinculadas ( Figura 1 ). 

**Figura 1:** - Correlación de Pearson entre variables numéricas (n = 124). Bucaramanga, Colombia, 2023

Nota: Experiencia en años

*H_Semana = Horas de trabajo a la semana

 En el análisis según las categorías se aprecia que las dimensiones tienen en su mayor porcentaje niveles de muy alto y alto de conocimiento, prevención y actitud, donde en conocimientos el 65,32% (81/124) con puntajes muy altos, el 33,06% (41/124) en altos y el restante 1,62% (2/124) con niveles muy bajo/bajo/medio, en la de prevención el 66,94% (82/124) con niveles altos, el 31,45% (39/124) con altos, y el 0,81% (2) con niveles muy bajo/bajo/medio, para la dimensión de actitud el 56,45% (70/124) con niveles muy altos, el 34,68% (43/124) con niveles altos y el 8,88% (11/124) con niveles muy bajo/bajo/medio. Para la dimensión de opinión, el 47,58% (59/124) con puntajes muy altos, el 48,39% (60/124) en altos, y el 4,04% (5/124) con niveles muy bajo/bajo/medio ( Figura 2 ). 

**Figura 2:** - Distribución porcentual de los niveles según las dimensiones del instrumento (n = 124). Bucaramanga, Colombia, 2023

 En el análisis múltiple se identifica que los enfermeros(as) que laboran en instituciones privadas presentan de forma significativa (valor p <0,05) puntajes en las categorías de muy altos en las diferentes dimensiones en comparación con los que se desempeñan en instituciones públicas, de otra parte, se parecía que el tener un nivel de especialización favorece también los puntajes muy altos. El sexo masculino se aprecia que en la dimensión de actitud se relacionan tener puntajes muy altos ( Tabla 2 ). En la edad se observó tendencia a tener menores puntajes en las dimensiones a medida que el rango de edad aumenta y esto de forma significativa en las dimensiones de conocimiento y prevención para la categoría de 31 a 35 años en comparación los de 25 a 30 años ( Tabla 2 ). 

**Tabla 2:** - *Odds Ratio* ajustado para estar en el nivel más alto en cada una de las dimensiones de la escala (n = 124). Santander, Colombia, 2023

**Característica**	**Conocimiento**	**Prevención**	**Actitud**	**Opinión**
Edad en años				
25-30	1	1	1	1
31-35	0,23 (0,07; 0,78)*	0,20 (0,05; 0,77)*	1,12 (0,38; 3,22)	1,18 (0,34; 4,01)
36-40	0,12 (0,01; 1,09)	0,21 (0,02; 2,16)	0,85 (0,12; 6,05)	0,26 (0,03; 2,18)
≥ 41 años	0,13 (0,00; 1,90)	0,06 (0,00; 1,13)	0,28 (0,02; 2,83)	0,85 (0,06; 10,78)
Sexo				
Femenino	1	1	1	1
Masculino	2,32 (0,73; 7,39)	2,73 (0,78; 9,55)	3,31 (1,18; 9,23)*	1,20 (0,41; 3,48)
Estado civil				
Soltero/a	1	1	1	1
Casado/a	0,93 (0,34; 2,53)	1,24 (0,43; 3,59)	1,01 (0,41; 2,48)	1,78 (0,61; 5,19)
Nivel educativo				
Profesional	1	1	1	1
Especialista	2,98 (0,79; 11,15)	2,36 (0,64; 8,63)	2,84 (0,89; 9,06)	7,60 (1,73; 33,23)^†^
Lugar de trabajo				
Público	1	1	1	1
Privado	3,92 (1,50; 10,25)	8,93 (3,12; 25,565)^‡^	2,77 (1,16; 6,58)*	19,65 (5,85; 65,94)^‡^

*Valor p <0.05; ^†^<0.01; ^‡^< 0.001

## Discusión

El presente estudió analizó la relación entre la conciencia, prevención, actitudes y percepción sobre el COVID-19 con las características sociodemográficas y laborales de los profesionales de enfermería, donde las dimensiones de conciencia y prevención presentaron porcentajes mayores al 90%. Esto refleja la mejora en conocimientos y el desarrollo de protocolos en nuevas enfermedades infecciosas, pues durante el periodo crítico de la pandemia, hubo la necesidad de avanzar en el manejo de la enfermedad^(27-29)^.

Lo anterior ha sido demostrado en diferentes estudios realizados en países protagonistas como China, donde han referenciado adecuadas actitudes y prácticas en el 97,40% y 92,70% respectivamente en los participantes^(30)^. Sin embargo, con relación a los conocimientos, solo el 6,40% obtuvieron un buen nivel, algo inquietante, pues el estudio fue llevado después de dos años del inicio de la pandemia. En lo correspondiente a la correlación entre los conocimientos, actitudes y prácticas se obtuvieron puntajes en el *r* entre 0,03 y 0,29, lo cual corresponde a puntajes más bajos que los reportados en el presente estudio^(30)^. Estas diferencias pudieran deberse a los esfuerzos y las diferentes estrategias utilizadas en los dos países en donde las diferencias culturales y los procesos educativos en salud son diferentes.

La correlación positiva fuerte identificada entre conciencia-prevención, conciencia-actitud y prevención-actitud con una *r* mayor a 0,50, así como entre los años de experiencia con la dimensión conciencia con una r=0,19 y los años de experiencia con la dimensión opinión con una *r*=0,19, se asemeja a lo descrito en un estudio realizado con 182 enfermeros(as) de UCIA en Jordania donde identificaron relación entre los años de experiencia con los conocimientos^(31)^. Así mismo, en Arabia Saudita se encontró una relación entre mejores puntajes de conocimiento con un nivel educativo superior, siendo los especialistas aquellos con mejores niveles^(32)^. Estos hallazgos evidencian el efecto y la necesidad de los programas de educación continuada para profesionales de enfermería^(33-34)^.

En lo relacionado con el tipo de institución en donde se labora, es decir, pública o privada, podría tener relación a la disponibilidad de información, protocolos y prácticas institucionales, formación y experiencia individual, entre otros aspectos. Sin embargo, es algo que debe seguir siendo evaluado, pues los estudios encontrados corresponden a diferentes contextos en donde la cultura y la política pueden variar de manera radical^(35-36)^.

En cuanto al sexo, se identificó que los participantes de sexo masculino presentaron mejores puntajes, en especial en la dimensión de actitud. Debe considerarse que esto podría deberse al número de enfermeros que participaron, que corresponde a un menor porcentaje de la muestra. En relación con las actitudes y sus diferencias entre os sexos se encontró un estudio acerca de las razones para cambiar su trabajo o área de desempeño y tenían que ver con razones principalmente de remuneración económica^(37)^.

Como aportes al conocimiento, en el presente estudio se identifica la validación y uso de un instrumento estructurado para Colombia, el cual permitió evaluar las dimensiones de conciencia, actitudes, prevención y prácticas para la COVID-19. Además, como fortaleza del estudio se reconoce el tamaño de muestra, así como el análisis múltiple.

Dentro de las limitaciones se pueden considerar la población que incluye solo personal de servicio de la UCIA y el tipo de muestreo, dado que el realizar un censo proporcionaría un mayor poder en los cálculos^(38)^, como también, el contexto y número significativo de pacientes en la UCIA respecto al momento de la pandemia y recolección de los datos, lo que determina una limitante del presente estudio para hacer la extrapolación de los resultados, Cabe destacar que la infección respiratoria por COVID-19 es una entidad patológica que aún está en proceso de estudio, lo que implica que el conocimiento sobre esta enfermedad está en constante evolución.

Para futuros estudios se puede explorar la relación entre niveles de las diferentes dimensiones de la escala con variables de ansiedad^(29)^, antecedentes patológicos personales y de familiares relacionados con la COVID-19^(31)^; por otro lado, es importante destacar la evolución rápida en todo lo relacionado con el COVID-19, donde se identifica la importancia de estar actualizándose para brindar cuidados, tener diagnósticos más precisos y tratamientos eficaces, eficientes y efectivos, así como medidas de fomento, promoción y prevención innovadoras^(39-42)^.

## Conclusión

Los niveles de conciencia, prevención, actitud y opinión alrededor del COVID-19 presentaron mejores puntajes cuando los profesionales se desempeñaban en instituciones de carácter privado y cuando el nivel educativo era más alto, además, en la dimensión de actitud se apreció mejores puntajes en participantes de género masculino.

# Translation: Conhecimento, atitudes, prevenção e opinião de profissionais de enfermagem frente à COVID-19: estudo analítico^*^

**DOI:** 10.1590/1518-8345.7412.4417

**Authors:** Claudia Consuelo Torres-Contreras, Moisés Alfonso Bravo-Gómez, Raquel Rivera-Carvajal, Mario Castillo-Blanco, Diana Isabel Cáceres-Rivera

**Affiliations:** 1 Universidad de Santander, Facultad Ciencias de la Salud, Instituto de Investigacion Masira, Bucaramanga, Santander, Colômbia.; 2 Asociación de Medicina Crítica y Cuidado Intensivo, Bucaramanga, Santander, Colômbia.; 3 Universidad Cooperativa de Colombia, Facultad de Enfermería, Bucaramanga, Santander, Colômbia.

**Keywords:** COVID-19, Aprendizagem, Unidades de Terapia Intensiva, Pesquisa em Enfermagem, Prática Avançada de Enfermagem, Enfermagem de Cuidados Críticos

## Abstract

**Objetivo:**

analisar a relação entre o conhecimento, a prevenção, as atitudes e a opinião dos profissionais de enfermagem de unidades de terapia intensiva de adultos em relação à COVID-19 e às suas características sociodemográficas e de trabalho.

**Método:**

estudo transversal analítico. Foram incluídos 124 profissionais de enfermagem que atuavam em unidades de terapia intensiva adulta após a pandemia de COVID-19. Para mensurar as variáveis foi utilizado o questionário *Awareness, Attitudes, Prevention and Perceptions of COVID-19 Outbreak among Nurses*. Para identificar diferenças entre os grupos foram utilizados os testes Kruskal-Wallis e Mann-Whitney U, correlação de Pearson e análise múltipla em regressão logística para cada dimensão.

**Resultados:**

os participantes com vínculo laboral em instituições privadas apresentaram tendência a melhores escores em conscientização OR=3,92(IC95%:1,50; 10,25), em prevenção OR=8,93(IC95%:3,12; 25,565), em atitude OR=2,77 (IC95%: 1,16; 6,58) e na percepção com OR=19,65 (IC95%: 5,85; 65,94). Na atitude, os participantes do sexo masculino apresentaram melhor resultado com OR=3,31 (IC95%: 1,18; 9,23) e em relação à percepção, quem apresentou os melhores resultados foram aqueles com pós-graduação como especialistas OR=7,60 (IC95%: 1,73; 33,23).

**Conclusão:**

trabalhar em instituição privada e possuir pós-graduação do tipo especialização estiveram relacionados a melhores pontuações nas dimensões da escala.

## Destaques

**(1)** Anos de experiência apresentaram correlação positiva com conhecimento.

**(2)** Foram obtidos elevados níveis de sensibilização, práticas, prevenção e opiniões.

**(3)** Profissionais com especialização apresentam pontuações mais altas no KAB COVID-19.

**(4)** Profissionais de enfermagem mais velhos tendem a ter pontuações mais baixas nas dimensões do instrumento (conhecimento, prevenção, atitude e opinião).

**(5)** O período pandêmico aprimorou conhecimentos e práticas em enfermagem.

## Introdução

Os profissionais de enfermagem desempenharam um papel crucial no combate à pandemia da COVID-19. Eles foram fundamentais no cuidado direto aos pacientes infectados, no apoio à implementação de medidas de prevenção, na educação comunitária e em muitos outros aspectos^(1)^. Seus conhecimentos, atitudes e opiniões evoluíram durante e após a pandemia graças à rápida expansão do conhecimento científico sobre o vírus e a doença^(2)^.

No que diz respeito ao conhecimento dos profissionais de saúde e da equipe de enfermagem, em particular, eles têm que se manter constantemente atualizados sobre protocolos de cuidados, melhores práticas de prevenção de infecções e novas pesquisas relacionadas à COVID-19^(3)^, incluindo a questão central da vacinação^(4)^. No que diz respeito à prevenção, têm estado envolvidos na implementação de medidas, como a utilização adequada de equipamentos de proteção individual (EPI) e a promoção da vacinação. Também tem sido evidente o trabalho na educação dos pacientes, familiares e comunidades sobre a importância dessas práticas para reduzir a propagação deste vírus e suas variantes^(5-8)^.

Nesse mesmo sentido, a pandemia gerou uma carga emocional considerável para os profissionais de enfermagem, tendo sido reconhecido seu elevado nível de comprometimento e dedicação apesar dos desafios físicos e emocionais envolvidos no trabalho em ambientes altamente estressantes e muitas vezes perigosos, sendo isso evidente no cuidado proporcionado pelos profissionais a esses pacientes e seus familiares^(9-12)^.

Por outro lado, alguns profissionais expressaram preocupações com a falta de recursos e apoio, bem como o esgotamento, como aspectos negativos persistentes após a pandemia^(13-14)^, enquanto outros destacaram a importância da solidariedade e do trabalho em equipe como lições aprendidas^(15)^.

As lições aprendidas com a gestão do SARS-COV-1 em 2003 sugerem que o conhecimento e as atitudes em relação às doenças infecciosas estão associados ao nível de pânico, uma emoção que pode complicar as tentativas de preveni-las e evitar a propagação de seu contágio^(16)^.

O modelo KAB (*Knowledgement, Attitudes, and Behavior*) é um modelo teórico em educação que explica como o conhecimento influencia as mudanças comportamentais e enfatiza que estas são produto da interação entre conhecimentos e atitudes. Esse modelo estabelece que o comportamento em saúde pode ser modificado por três processos contínuos de mudança: aquisição de conhecimentos, formação de crenças e desenvolvimento de comportamentos^(6,17)^.

Atitudes, conhecimentos e práticas podem desempenhar um papel importante na forma como as medidas de controle são aceitas para prevenir uma doença infecciosa e sua propagação, e na adesão aos cuidados em geral, como é o caso das medidas para a COVID-19^(18)^. Adicionalmente, é necessário considerar que o confinamento provocou alterações comportamentais, somáticas e afetivas nos trabalhadores de enfermagem, frente às quais a avaliação dos processos de aprendizagem, conhecimento e exercício da profissão assumem cada vez mais relevância^(19)^.

Os profissionais de enfermagem podem ter opiniões diferentes em relação às doenças infecciosas e, portanto, podem aplicar diferentes estratégias de manejo clínico, levando a diferentes resultados para os pacientes^(20)^. Por outro lado, as práticas são comportamentos durante a interação com os pacientes para a prevenção e o cuidado àqueles que receberam esse diagnóstico.

Por esse motivo, a contribuição dos profissionais de enfermagem durante e após a pandemia tem sido essencial para combater e prevenir a propagação do vírus e suas variantes, bem como para prestar cuidados de qualidade às pessoas afetadas por esta doença no curto e longo prazos^(16-18)^. Assim, a equipe de pesquisa se propôs a analisar a relação entre o conhecimento, a prevenção, as atitudes e a opinião dos profissionais de enfermagem de unidades de terapia intensiva de adultos, com utilização do instrumento *Awareness, Attitudes, Prevention and Perceptions of COVID-19 Outbreak among Nurses* ^(21)^ em relação à COVID-19 e às suas características sociodemográficas e de trabalho.

## Método

### Desenho do estudo

Estudo observacional, transversal e analítico com informações de profissionais de enfermagem que trabalharam em Unidades de Terapia Intensiva de Adulto (UTIA) durante 2023 em Santander, Colômbia.

### População e amostra

O tamanho amostral necessário estipulado foi de 124 enfermeiros, considerando um aumento de 15% devido a possíveis ausências de respostas, admitindo-se um erro alfa de 0,05; uma potência de 0,80 e efeito de delineamento 1. Tomou-se como base os percentuais de níveis baixos reportados para as dimensões, onde a dimensão prevenção reporta 7,6% e seria a que requer o maior tamanho amostral, conforme publicado por estudo realizado na Arábia Saudita em 2020^(21)^.

### Instrumentos de estudo

Foi utilizado o Instrumento *Awareness, Attitudes, Prevention and Perceptions of COVID-19 Outbreak among Nurses* desenvolvido por Al-Dossary, et al.^(19)^, composto por 4 seções: conhecimento, prevenção, atitudes e opinião, com 63 itens com alfa de Cronbach relatado pelo autor de 0,73 a 0,97 nas dimensões. Este instrumento identifica níveis como baixo, médio e alto em cada uma de suas dimensões, com base em pontuações em escala do tipo Likert (1= Discordo totalmente a 5= Concordo totalmente). Para o presente estudo obteve-se um alfa de Cronbach na dimensão conhecimento de 0,90, na dimensão prevenção de 0,96, na dimensão atitude de 0,80 e na dimensão opinião de 0,83.

O instrumento foi traduzido e retrotraduzido, a validação de face e de conteúdo do questionário foi realizada com o conceito de 11 especialistas selecionados segundo os critérios do modelo Fehring^(22)^ com avaliação de clareza, precisão, pertinência e relevância, obtendo-se uma pontuação de Índice de Validade de Conteúdo (IVC) superior a 0,8 em 55 dos 63 itens. Os itens com IVC menor do que 0,8 corresponderam a:

*Dimensão conhecimento*: item 5. A COVID-19 é transmitida por meio do contato próximo com animais infectados; Item 13. A COVID-19 apresenta mortalidade menor que a infecção respiratória H1N1; Item 19. A COVID-19 pode ser transmitida pela ingestão de carne crua ou mal cozida ou de órgãos de animais; Item 23. Atualmente não há vacina disponível contra a COVID-19; Item 26. As estratégias terapêuticas atuais para tratar a infecção por COVID-19 são apenas preventivas e de suporte; Item 30. Pessoas que tiveram contato próximo com paciente confirmado positivo para COVID-19 durante os 14 dias anteriores ao início dos sintomas deverão ser colocadas em quarentena e submetidas a teste diagnóstico para COVID-19; item 32. Caso o resultado do teste seja positivo, recomenda-se que o teste seja repetido para verificação.

*Dimensão prevenção*: item 10. Somente casos suspeitos de pacientes com COVID-19 deverão ser mantidos em quarentena.

*Dimensão atitude*: item 6. Ter compensação monetária do governo ou do meu local de trabalho reduz minha ansiedade quanto ao risco de contrair a infecção.

Os autores da validação do instrumento determinaram excluir os itens 5 e 19 da dimensão conhecimento, o item 10 da dimensão prevenção e o item 6 da dimensão atitude. Os demais itens da dimensão conhecimento ficaram para análise e os escores dos itens 13, 23 e 32 foram revertidos, respectivamente. O instrumento validado ficou, então, com 36 itens na dimensão conhecimento, 13 na prevenção, 5 na atitude e 4 na opinião, totalizando 58 itens.

Os níveis são apresentados com base nas médias das pontuações da escala Likert, onde as pontuações de 1 a 1,79 são muito baixas, entre 1,8 a 2,59 são baixas, entre 2,6 a 3,39 são médias, entre 3,4 a 4,19 altas e de 4,2 a 5 muito alto^(23)^.

### Coleta de dados

Os dados foram coletados de fevereiro a maio de 2023, com a solicitação submetida *online*. Com informações dos bancos de dados da associação de medicina crítica de terapia intensiva de Santander, foi realizada uma amostragem estratificada selecionando-se profissionais de enfermagem que atualmente trabalhassem nas unidades de terapia intensiva que atendiam pacientes com diagnóstico positivo para COVID-19 nos municípios de Bucaramanga, Floridablanca, Piedecuesta, San Gil, Socorro e Barrancabermeja. Como critérios de inclusão, considerou-se que atuassem há mais de 3 meses na UTI onde são atendidos pacientes com diagnóstico de COVID-19; deficiência física ou não ter acesso à tecnologia para responder ao questionário foram considerados critérios de exclusão.

### Análise de dados

Na análise, as variáveis de medida qualitativa foram descritas com frequências absolutas e relativas, as variáveis contínuas descritas com medidas de tendência central e dispersão de acordo com a distribuição, com distribuição normal foram apresentadas a média e o desvio-padrão, e as variáveis não paramétricas foram apresentadas com medianas e intervalos interquartis; para verificar a distribuição utilizou-se o teste de Shapiro-Francia.

Foi realizada análise bivariada com os escores médios em cada uma das dimensões, para comparação das características dos participantes, utilizando-se os respectivos testes estatísticos de Kruskal-Wallis e teste U de Mann-Whitney. A análise de correlação foi realizada com a estatística de Pearson para as variáveis numéricas após avaliação da distribuição normal e foi gerado um gráfico no *software R.* Para a análise múltipla foi utilizada a regressão logística para determinar a probabilidade de estar no nível mais alto na comparação. Com os demais níveis foi realizada uma avaliação do ajuste do modelo. Os cálculos foram realizados no *software* estatístico *Stata v17*. O banco de dados e a análise do processo de validação do instrumento e dos resultados apresentados foram carregados no *Mendeley Data* ^(24)^.

### Aspectos éticos

O estudo baseou-se na Resolução 8.430 de 1993 do Ministério da Saúde da Colômbia^(25)^ e na Declaração de Helsinki promulgada pela Associação Médica Mundial em 2000^(26)^. A pesquisa foi considerada um estudo livre de risco. Dentre os princípios éticos da pesquisa foram levados em consideração a autonomia, a confidencialidade, a beneficência e a não maleficência e foi solicitado o consentimento informado dos participantes, que foi vinculado no formulário *online* anterior ao instrumento. No documento, o objetivo da pesquisa foi explicado aos participantes e destacou-se que sua participação e desistência seriam voluntárias. O presente estudo foi aprovado pelos comitês de bioética da Universidade de Santander, com registro nº 004 de 15 de fevereiro de 2022.

## Resultados

Quanto às características dos 124 participantes do estudo, foi observado, na maioria, uma população jovem com 46,77% (58/124) na faixa etária entre 25 e 30 anos, seguida dos participantes entre 31 e 35 anos com 30,65% (38/124); os maiores de 46 anos correspondiam a 3,23% (4/124); 76,61% (95/124) eram do sexo feminino e 23,39% do sexo masculino (29/124); o estado civil de maior prevalência foi solteiro com 69,35% (86/124), seguido dos casados com 29,03% (36/124). Nos anos de experiência profissional na UTIA identificou-se mediana de 7 (RI: 4; 10,5) anos e número de horas de trabalho por dia com mediana de 12 (RI: 12; 12). Quanto à escolaridade, identificou-se que 77,42% (96/124) correspondiam a profissionais com graduação, 18,55% (23/124) especialistas e 4,03% (5/124) a profissionais com mestrado. Com relação aos vínculos, 42,98% (49/124) eram ligados a instituições de saúde do setor público e 57,02% (65/124) com o setor privado. A dimensão com melhor pontuação mediana foi a prevenção, com mediana de 4,73 (4,07; 5) e a menor foi a opinião, com mediana de 4 (4; 4,75) (Tabela 1).

Ao comparar as medianas das pontuações das dimensões segundo características sociodemográficas e ocupações, verificam-se diferenças com os valores das dimensões conhecimento, prevenção e opinião, em que os participantes que trabalhavam em instituições privadas apresentavam medianas superiores aos de áreas públicas (p<0,05). Em prevenção e atitude, os participantes do sexo masculino apresentaram medianas mais altas em comparação aos participantes do sexo feminino (Tabela 1).

**Tabela 1:** - Características dos participantes. Medianas e intervalos interquartis segundo dimensões (n = 124). Santander, Colômbia, 2023

**Recurso**	**%(n)**	**Conhecimento**			**Prevenção**			**Atitude**			**Opinião**		
	100(124)	4,36	(4; 4,58)		4,73	(4,07; 5)		4,20	(4; 5)		4	(4; 4,75)	
Idade em anos				0,5795			0,6931			0,8182			0,1364
25-30	46,77(58)	4,36	(4,04; 4,58)		4,69	(4,07; 5)		4,20	(3,60; 5)		4	(3,75; 4,50)	
31-35	30,65(38)	4,27	(3,91; 4,55)		4,80	(4; 5)		4,40	(4; 4,80)		4,37	(4; 4,75)	
36-40	8,87(11)	4,47	(3,88; 4,63)		5	(4,07; 5)		4,60	(3,20; 5)		4	(3,75; 5)	
41-45	10,48(13)	4,38	(3,94; 4,55)		4,38	(4,07; 4,92)		4	(4; 4,40)		4,25	(4; 5)	
> 46	3.23(4)	4,47	(4,31; 4,68)		4,92	(4,61; 5)		4,60	(4; 5)		4,85	(4,12; 5)	
Sexo				0,2397			**0,0016**			**0,0525**			0,2965
Feminino	76,61(95)	4,29	(3,97; 4,58)		4,69	(4,62; 4,92)		4,20	(4; 4,80)		4	(4; 4,75)	
Masculino	23,39(29)	4,50	(4,22; 4,61)		5	(4,38; 5)		4,80	(4,20; 5)		4,50	(4; 5)	
Estado civil				0,9508			0,0941			0,2618			0,1399
Solteiro	69,35(86)	4,36	(3,97; 4,58)		4,73	(4,07; 5)		4,20	(4; 5)		4	(4; 4,50)	
Casado	29.03(36)	4,37	(4,02; 4,58)		4,80	(4,15; 5)		4,20	(4; 4,80)		4,50	(4; 5)	
Divorciado	1,61(2)	16,30	(4,22; 4,38)		2,57	(1,15; 4)		2,90	(1,80; 4)		4,37	(3,75; 5)	
Nível educacional				0,3186			0,5817			0,2343			0,2767
Profissional	77,42(96)	4,27	(3,97; 4,58)		4,69	(4,07; 5)		4,20	(4; 5)		4	(4; 4,75)	
Especialista	18,55(23)	4,50	(4,25; 4,63)		4,84	(4,15; 5)		4,60	(4; 5)		4,25	(4; 4,75)	
Mestrado	4.03(5)	4,37	(4,34; 4,41)		4,92	(4,69; 4,92)		4,20	(3,60; 4,60)		5	(4,5; 5)	
Local de trabalho				**0,0199**			**0,0166**			0,4375			**<0,0001**
Público	42,98(49)	4,22	(3,94; 4,55)		4,07	(4; 5)		4	(4; 4,80)		4	(3,75;4)	
Privado	57,02(65)	4,41	(4,22; 4,61)		4,76	(4,38; 5)		4,20	(4; 4,80)		4,50	(4; 5)	

Nota: valor p: teste U de Kruskal-Wallis e Mann-Whitney

Na análise de correlação para as variáveis numéricas, constatou-se que os anos de experiência apresentaram correlação significativa com o número de horas trabalhadas por dia e a dimensão conhecimento. Também foi identificada correlação entre os escores das dimensões do instrumento, onde os mais fortes corresponderam aos escores conhecimento-prevenção, prevenção-atitude com correlações superiores a 0,64, (valor P <0,0001). A experiência em anos apresentou correlação significativa mas de baixa intensidade com conhecimento-opinião, de 0,19 e 0,22 respectivamente (valor de p = 0,0398 e 0,0159); as horas de trabalho semanais não se correlacionaram significativamente com as variáveis vinculadas (Figura 1).

**Figura 1:** - Correlação de Pearson entre variáveis numéricas (n = 124). Bucaramanga, Colômbia, 2023

Nota: Experiência expressa em anos

*H_Semana = Horas de trabalho por semana

Na análise segundo as categorias, percebeu-se que as dimensões apresentaram os maiores percentuais de níveis muito alto e alto de conhecimento, prevenção e atitude, onde em conhecimento 65,32% (81/124) foram pontuações muito altas, 33,06 % (41/124) foram de nível alto e os restantes 1,62% (2/124) com níveis muito baixo/baixo/médio. Na categoria prevenção, 66,94% (82/124) foram níveis elevados, 31,45% (39/124) com níveis altos e 0,81% (2) com níveis muito baixo/baixo/médio. Para a dimensão atitude, 56,45% (70/124) com níveis muito altos, os 34,68% (43/124) com níveis altos e 8,88% (11 /124) com níveis muito baixos/baixos/médios. Para a dimensão opinião, 47,58% (59/124) com pontuações muito altas, 48,39% (60/124) com pontuações altas e 4,04% (5/124) com níveis muito baixos/médios (Figura 2).

**Figura 2:** - Distribuição percentual dos níveis segundo as dimensões do instrumento (n = 124). Bucaramanga, Colômbia, 2023

Na análise múltipla, identifica-se que os enfermeiros que trabalhavam em instituições privadas apresentaram pontuações significativas (p valor <0,05) nas categorias muito altas nas diferentes dimensões, em comparação com os que trabalhavam em instituições públicas. Ter um nível de especialização também favoreceu pontuações muito altas. Para o sexo masculino, verifica-se que a dimensão atitude está relacionada com pontuações muito elevadas (Tabela 2). Em termos de idade, observou-se uma tendência de menores pontuações nas dimensões à medida que a faixa etária aumentava, e isso foi significativo nas dimensões de conhecimento e prevenção para a categoria de 31 a 35 anos, em comparação com aqueles de 25 a 30 anos (Tabela 2).

**Tabela 2:** - *Odds Ratio* ajustado para estar no nível mais alto em cada uma das dimensões da escala (n = 124). Santander, Colômbia, 2023

**Recurso**	**Conhecimento**	**Prevenção**	**Atitude**	**Opinião**
Idade em anos				
25-30	1	1	1	1
31-35	0,23 (0,07, 0,78)*	0,20 (0,05, 0,77)*	1,12 (0,38, 3,22)	1,18 (0,34, 4,01)
36-40	0,12 (0,01, 1,09)	0,21 (0,02, 2,16)	0,85 (0,12, 6,05)	0,26 (0,03, 2,18)
≥ 41 anos	0,13 (0,00, 1,90)	0,06 (0,00, 1,13)	0,28 (0,02, 2,83)	0,85 (0,06, 10,78)
Sexo				
Feminino	1	1	1	1
Masculino	2,32 (0,73, 7,39)	2,73 (0,78, 9,55)	3,31 (1,18; 9,23)*	1,20 (0,41, 3,48)
Estado civil				
Solteiro	1	1	1	1
Casado	0,93 (0,34, 2,53)	1,24 (0,43, 3,59)	1,01 (0,41, 2,48)	1,78 (0,61, 5,19)
Nível educacional				
Profissional	1	1	1	1
Especialista	2,98 (0,79, 11,15)	2,36 (0,64, 8,63)	2,84 (0,89, 9,06)	7,60 (1,73; 33,23)^†^
Local de trabalho				
Público	1	1	1	1
Privado	3,92 (1,50, 10,25)	8,93 (3,12; 25,565)^‡^	2,77 (1,16; 6,58)*	19,65 (5,85; 65,94)^‡^

*Valor de p<0,05; ^†^<0,01; ^‡^< 0,001

## Discussão

Este estudo analisou a relação entre conscientização, prevenção, atitudes e percepção sobre a COVID-19 e as características sociodemográficas e laborais dos profissionais de enfermagem, em que as dimensões de conscientização e prevenção apresentaram percentuais superiores a 90%. Tal fato reflete a melhoria do conhecimento e o desenvolvimento de protocolos para tratamento de novas doenças infecciosas, uma vez que durante o período crítico da pandemia foi necessário avançar no manejo da doença^(27-29)^.

O exposto foi demonstrado em diversos estudos realizados em países líderes como a China, em que atitudes e práticas adequadas foram relatadas, respectivamente, em 97,40% e 92,70% dos participantes^(30)^. Em relação ao conhecimento, apenas 6,40% obtiveram nível bom, algo preocupante, já que o estudo foi realizado dois anos após o início da pandemia. Quanto à correlação entre conhecimentos, atitudes e práticas, foram obtidos escores *r* entre 0,03 e 0,29, o que corresponde a escores inferiores aos relatados no presente estudo^(30)^. Estas diferenças podem dever-se aos esforços e às diferentes estratégias utilizadas nos dois países, onde as diferenças culturais e os processos de educação para a saúde são diferentes.

A forte correlação positiva identificada entre conscientização-prevenção, conscientização-atitude e prevenção-atitude com *r* superior a 0,50, bem como entre os anos de experiência com a dimensão conscientização com r=0,19 e os anos de experiência com a dimensão opinião com *r* =0,19, é semelhante ao descrito em estudo realizado com 182 enfermeiros de UTIA na Jordânia, onde identificou-se relação entre anos de experiência e conhecimento^(31)^. Da mesma forma, na Arábia Saudita, foi encontrada relação entre melhores escores de conhecimento com maior nível educacional, sendo os especialistas aqueles com melhores níveis^(32)^. Esses achados mostram o efeito e a necessidade de programas de educação continuada para profissionais de enfermagem^(33-34)^.

Quanto ao tipo de instituição onde se trabalha, ou seja, pública ou privada, pode haver relação com a disponibilidade de informações, protocolos e práticas institucionais, formação e experiência individual, entre outros aspectos. Contudo, é algo que deve continuar a ser avaliado, uma vez que os estudos encontrados correspondem a diferentes contextos onde a cultura e a política podem variar radicalmente^(35-36)^.

Em relação ao sexo, identificou-se que os participantes do sexo masculino apresentaram melhores pontuações, principalmente na dimensão atitude. Deve-se considerar que isso pode ser devido ao número de enfermeiros participantes, o que corresponde a um percentual menor da amostra. Em relação às atitudes e suas diferenças entre os sexos, foi encontrado um estudo sobre os motivos de mudança de emprego ou área de atuação, que tinham a ver com motivos principalmente de remuneração^(37)^.

Como contribuições ao conhecimento, este estudo identifica a validação e utilização de um instrumento estruturado para a Colômbia, o que permitiu avaliar as dimensões de conscientização, atitudes, prevenção e práticas em relação à COVID-19. Além disso, o ponto forte do estudo é o tamanho da amostra, bem como a análise múltipla.

Dentre as limitações, podemos considerar a população que inclui apenas funcionários do serviço da UCIA e o tipo de amostragem, visto que a realização de um censo daria maior poder nos cálculos^(38)^, bem como o contexto e número significativo de pacientes internados na UTI com relação ao momento da pandemia e coleta de dados, o que determina uma limitação do presente estudo para extrapolar os resultados. Cabe ressaltar que a infecção respiratória por COVID-19 é uma entidade patológica que ainda está em processo de estudo, o que implica que o conhecimento sobre esta doença está em constante evolução.

Para estudos futuros, pode-se explorar a relação entre os níveis das diferentes dimensões da escala com variáveis de ansiedade^(29)^ e história patológica pessoal e familiar relacionada à COVID-19^(31)^; por outro lado, é importante destacar a rápida evolução em tudo relacionado ao COVID-19, contexto em que é importante estar atualizado para prestar cuidados, ter diagnósticos mais precisos e tratamentos eficazes, eficientes e efetivos, bem como medidas de fomento. promoção e prevenção inovadoras^(39-42)^.

## Conclusão

Os níveis de conscientização, prevenção, atitude e opinião relacionadas à COVID-19 apresentaram melhores pontuações quando os profissionais trabalhavam em instituições privadas e quando o nível de escolaridade era maior. Além disso, na dimensão atitude, foram observadas melhores pontuações nos participantes do sexo masculino.
